# Corrosion Passivation in Simulated Body Fluid of Ti-Zr-Ta-xSn Alloys as Biomedical Materials

**DOI:** 10.3390/ma16134603

**Published:** 2023-06-26

**Authors:** El-Sayed M. Sherif, Yassir A. Bahri, Hamad F. Alharbi, Muhammad Farzik Ijaz

**Affiliations:** 1 Center of Excellence for Research in Engineering Materials (CEREM), Deanship of Scientific Research, King Saud University, Riyadh 11421, Saudi Arabia; 2 Mechanical Engineering Department, Collage of Engineering, King Saud University, Al-Riyadh 11421, Saudi Arabia

**Keywords:** Ti-based alloys, corrosion, Sn content, simulated body fluid, electrochemical techniques, spectroscopic analysis

## Abstract

The powder metallurgy method was used to manufacture three Ti-based alloys: Ti-15%Zr-2%Ta-4%Sn (Ti-Zr-Ta-4Sn), Ti-15%Zr-2%Ta-6%Sn (Ti-Zr-Ta-6Sn), and Ti-15%Zr-2%Ta-8%Sn (Ti-Zr-Ta-8Sn). Electrochemical measurements and surface analyses were used to determine the effect of Sn concentration on the corrosion of these alloys after exposure to a simulated body fluid (SBF) solution for 1 h and 72 h. It was found that the passivation of the alloy surface significantly increased when the Sn content increased from 4% to 6% and then to 8%, which led to a significant reduction in corrosion. The impedance spectra derived from the Nyquist graphs also explained how the addition of Sn significantly improved the alloys’ polarization resistances. According to the change in the chronoamperometric current at an applied anodic potential over time, the increase in Sn content within the alloy significantly reduced the currents over time, indicating that the uniform and pitting corrosion were greatly decreased. The formation of an oxide layer (TiO_2_), which was demonstrated by the surface morphology of the alloys after exposure to SBF solution for 72 h and corrosion at 400 mV (Ag/AgCl) for 60 min, was supported by the profile analysis obtained by an X-ray spectroscopy analyzer. It was clear from all of the findings that the tested alloys have a remarkable improvement in resistance to corrosivity when the Sn content was increased to 8%.

## 1. Introduction

The necessity for substituting hips and other human body parts has significantly increased the need for biomedical implants today. Every year, about one million people undergo hip replacements. From this perspective, it is essential to choose high-quality materials for such applications since they have high corrosion resistance and excellent mechanical and biocompatible features [1,2,3,4,5]. Ti-based alloys are significant for many different applications in addition to biomedicine, for example, in the automotive and aerospace industries. Their outstanding biocompatibility, extraordinary corrosion resistance, and high mechanical strength allow these alloys to be successfully used in biomedical applications [5,6,7,8,9,10].

Although the titanium–aluminum–vanadium (Ti-6Al-4V) alloy has long been employed in such applications, the V and Al released from the alloy could have harmful effects [7,8,9,10,11,12]. Al^+3^ and V^+5^ are released as free radicals, interact with the bloodstream, and possibly trigger harmful responses in humans, which increases the risk of developing Alzheimer’s disease [13,14,15,16,17]. For this reason, Al and V should be replaced with other noble metals in order to prevent this undesirable impact. Lin et al. [18] studied the implantation of varying ratios of Ta, Hf, and Zr to create various series of Ti-Ta-Hf-Zr alloys. In addition to having an exceptionally high elastic admissible strain, Ti-Ta-Hf-Zr alloys were discovered to have high mechanical strength and good biocompatibility [18]. The alloying of Zr, Ta, Nb, and Sn with Ti to create various alloys for use in diverse applications, including the bone response to a Ti-Ta-Nb-Zr alloy when it is present inside the human body, has been reported by Grandin et al. [19], Hu et al. [20], Tahara et al. [21], Kurthz et al. [22], and Stenlund et al. [23]. There have also been reports that Ti-39Nb and Ti-6Al-7Nb combinations are highly corrosion-resistant alloys [24,25,26].

Zirconium (Zr) has been reported to have great mechanical strength and good corrosion resistance, making it the ideal material for biomedical implants [27,28,29]. Furthermore, because tantalum (Ta) does not irritate the body, it is used to make surgical tools like surgical sutures, prosthetic joints, and cranial plates [30,31]. Both Ta and Zr have similar biocompatibility to titanium, and the changes in their amounts have little impact on the density of cell adhesions when Ti is present. Also, Ti-Zr-Mo alloy production has reportedly been used in medicinal applications [32]. Moreover, the effect of adding Sn on the mechanical properties of some Ti-based alloys, such as Ti-Al alloys [33], has been reported, and it was found that the presence of 1.85 at% improves the ductility of the alloys. Keshtta1 and Gepreel [34] have also reported the effect of various Sn additions (0, 1.5, and 3 at%) on the mechanical properties of the Ti-17Nb-6Ta alloy and found that adding Sn at 1.5% increases the hardness of the alloy, but that increasing it to 3% has a negligible effect apart from increasing other mechanical features. The influence of 5 at% Sn addition on the mechanical and surface properties of different Ti-Nb alloys was also reported by Torres-Sánchez et al. [35], who found that the presence of Sn at this at% enhances the passivation of the surface via oxide layer formation.

It is worth mentioning that the effect of Zr and Sn addition on the microstructure of Ti-Nb-Fe alloys was also reported by Dal Bó et al. [36], and it was found that Sn increases the yield strength, whereas Zr reduces the elastic modulus. The effect of adding Sn on the mechanical properties of our Ti-Zr-Ta alloy has also been reported, and it was found that Sn has a great impact on improving the mechanical properties of the alloy [7]; this was also reported by Ion et al. [37] for the Ti-Hf-Mo-Sn alloy. In addition to the great biocompatibility of these alloys, they have excellent corrosion resistance to most corrosive media, including human body fluids and saliva. From this point of view, this study aims to manufacture different Ti-Zr-Ta alloys with different weight % of tin (Sn). The aim is extended to report the effect of changing the Sn content from 4% to 6% and further to 8% on the corrosion behavior of these alloys in a simulated body fluid solution. The objectives of this work can be achieved by using polarization and EIS, and by changing the current as a function of time at a defined active anodic voltage.

## 2. Materials and Methods

### 2.1. Chemicals and Materials

The test solution was SBF, which was purchased from Life technologies™ (Warrington, UK). SBF consists of KCl, Na_2_SO_4_, NaCl, NaHCO_3_, K_2_HPO_4,_ MgCl_2_.6H_2_O, and CaCl_2_ [38]. The chemical substances used to fabricate the different alloys, Ti-15Zr-Ta-xSn, were the powders of >99% purity Ti (5–7 μm particle size (PS)), ~99% purity Zr (10 μm PS), >99% purity Ta (10 μm PS), and 99% purity Sn (10 μm PS). All of these powders were supplied by Nanochemazone, Canada. The alloys used were manufactured by mixing the corresponding weight % (wt.%) of their powders using a high-energy ball mill. The mixed powders were then sintered inside a high-frequency heat induction furnace. The detailed procedures for the fabrication of the test alloys can be found in [39].

### 2.2. Electrochemical Cell and Electrochemical Techniques

A computer-controlled Autolab (Metrohm, Amsterdam, The Netherlands) was utilized for the electrochemical experiments. A three-electrode electrochemical cell that accommodates 150 mL solution was used. The Ag/AgCl, a platinum sheet, and the tested alloys were used as the reference, counter, and working electrodes, respectively. The cyclic potentiodynamic polarization results were collected in the forward and backward directions over a potential range from −1000 to 400 mV; the scan rate for both directions was 1.667 mV (Ag/AgCl) per second. The electrochemical impedance data were gathered from the free (corrosion) potential within 100 kHz to 0.10 Hz as a frequency range. The curves of the change in the chronoamperometric current with time at 400 mV (as a constant voltage) were obtained for the different alloys over 60 min of the potential application. All the electrochemical experiments were performed at room temperature. A JSM-7400F machine (SEM, JEOL microscopy model Tokyo, Japan) with an energy-dispersive X-ray (EDX) attached unit was employed to report the morphology of the surfaces along with their EDX profiles.

## 3. Results and Discussion

### 3.1. CPP Measurements

**Figure 1:**
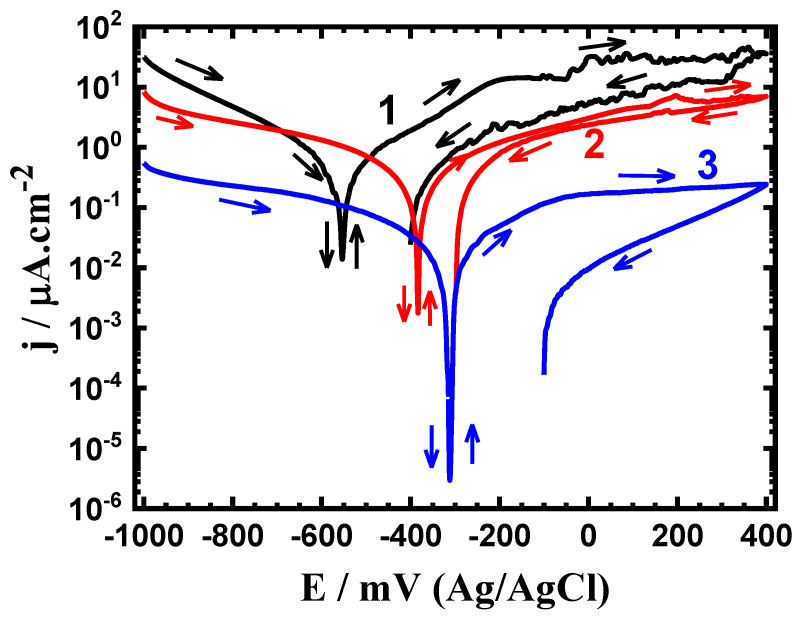
CPP profiles after one hour in SBF for (1) Ti-Zr-Ta-4Sn, (2) Ti-Zr-Ta-6Sn, and (3) Ti-Zr-Ta-8Sn, respectively.

**Figure 2:**
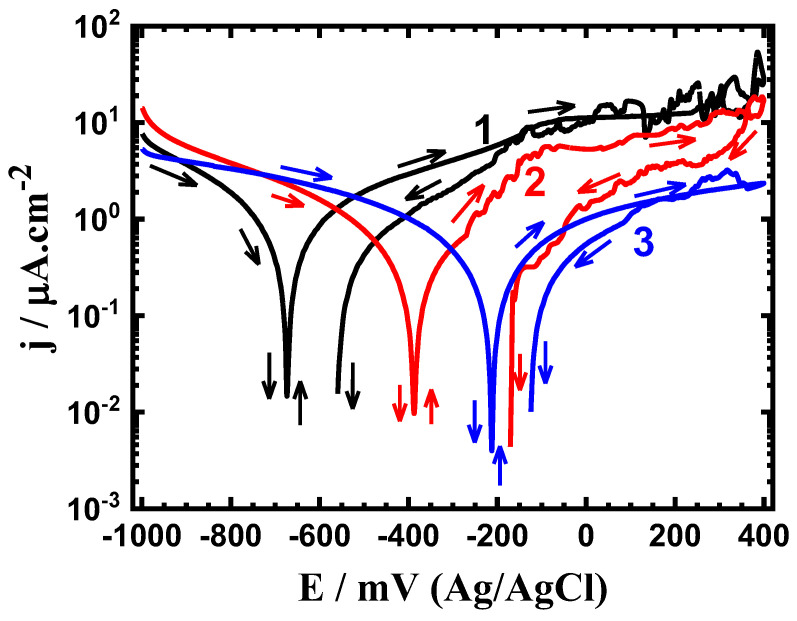
CPP profiles after 72 h in SBF for (1) Ti-Zr-Ta-4Sn, (2) Ti-Zr-Ta-6Sn, and (3) Ti-Zr-Ta-8Sn, respectively.

Cyclic potentiodynamic polarization (CPP) experiments were carried out to ascertain the effects of the Sn content and exposure duration on the electrochemical behavior of the sintered Ti-base alloys. The CPP curves for the sintered (1) Ti-Zr-Ta-4Sn, (2) Ti-Zr-Ta-6Sn, and (3) Ti-Zr-Ta-8Sn alloys after being submerged in SBF for one hour are shown in Figure 1. Additionally, other CPP curves were collected after 72 h, and the resulting plots are shown in Figure 2. The Tafel slopes of the cathode (βc) anode (βa), corrosion potential (E_Corr_), corrosion current density (j_Corr_), polarization resistance (R_P_), and corrosion rate (R_Corr_) that were obtained from the CPP data are shown in Table 1. The values of βc and βa were calculated from the slopes of the cathodic and anodic currents, respectively. The values of E_Corr_ and j_Corr_ were obtained from the intersection of the lines drawn next to the anodic and cathodic Tafel current regions. The values of Rp and R_Corr_ were calculated from the values of βc, βa, and j_Corr_ using the same procedures and equations described in [39].

Figure 1 and Figure 2 show that applying potential in a very negative orientation causes the current in the cathode to decrease due to the reduction of oxygen. Several studies [38,39,40,41] have shown that metals and alloys in a neutral solution such as a simulated body fluid undergo cathodic reduction of oxygen.

2H_2_O + O_2_ + 4e^−^ = 4OH^−^ (1)

The increase of Sn content from 4% to 6% and further to 8% within the alloy is seen to decrease the intensity of this cathodic reaction by decreasing the cathodic current. This decrease in the cathodic reaction shifts E_Corr_ to less negative values and allows j_Corr_ to record lower values as can be seen in Table 1.

Scanning the potential in the anodic side causes an abrupt rise in currents, which marks the beginning of the anodic reaction of the sintered alloys, as shown in Figure 1. The increase in the current occurs as a consequence of the dissolution of the oxide layer that may form during the early stages of the anodic potential application. Because Sn can passivate Ti-Zr-Ta alloy in the SBF electrolytic solution, a rise in the Sn content significantly reduces the anodic currents. In addition, all sintered alloys showed lower backward current values obtained when the potential was reversed from 400 mV to negative values. This proves that pitting corrosion does not occur under these circumstances [39,41].

It is also observed that increasing the Sn content when the time of exposure was increased to 72 h, as shown in Figure 2, greatly affects the corrosion of the tested alloys. Here, it is seen that the cathodic and anodic reactions are accelerated by rinsing the alloys in the SBF solution for 72 h. The greater recorded current values compared to those plotted after a one-hour immersion (Figure 1) served as evidence for this observation. Here, the values of j_Corr_ are significantly reduced as Sn% increases, but at greater values than those shown in Figure 1. As the immersion time was extended, the values of R_P_ also dropped and the values of R_Corr_ rose.

### 3.2. CCt Measurements

**Figure 3:**
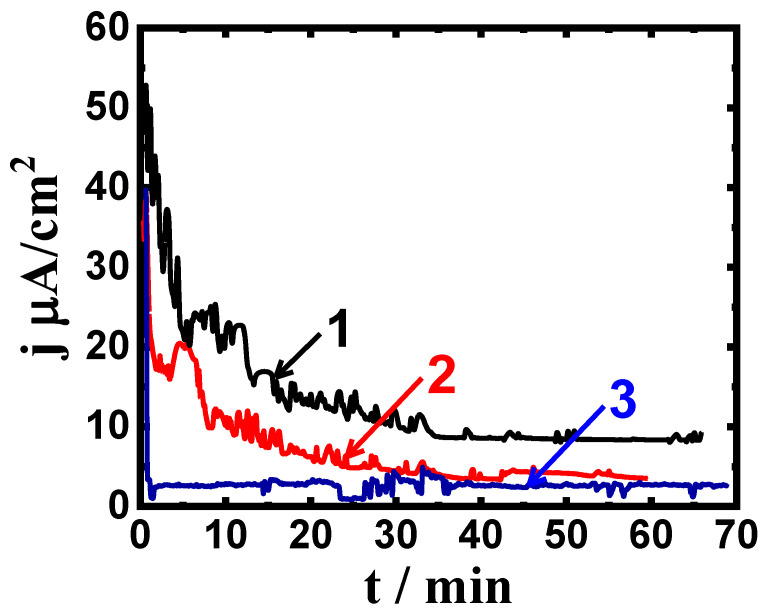
CCt profiles that were obtained for (1) Ti-Zr-Ta-4Sn, (2) Ti-Zr-Ta-6Sn, and (3) Ti-Zr-Ta-8Sn at 400 mV (Ag/AgCl) after being submerged in SBF for one hour.

**Figure 4:**
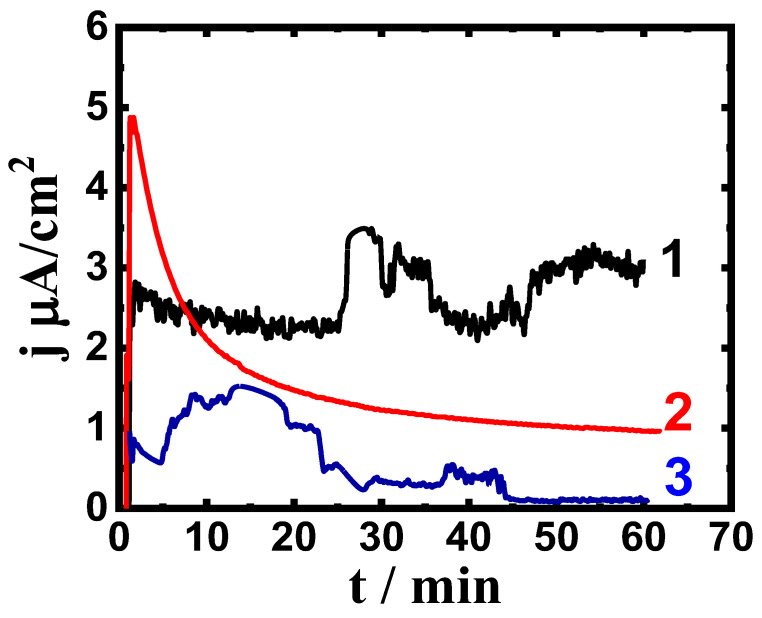
CCt profiles that were obtained for (1) Ti-Zr-Ta-4Sn, (2) Ti-Zr-Ta-6Sn, and (3) Ti-Zr-Ta-8Sn at 400 mV (Ag/AgCl) after being submerged in SBF for 72 h.

Chronoamperometric current with time (CCt) measurements have been utilized to report the corrosion of various metallic structures at a fixed anodic potential value [38,39,41]. Figure 3 represents the CCt curves for (1) Ti-Zr-Ta-4Sn, (2) Ti-Zr-Ta-6Sn, and (3) Ti-Zr-Ta- 8Sn that were obtained at 400 mV after one hour in the SBF solution, while Figure 4 displays the CCt curves that were obtained at the same value of potential for the same alloys after exposure for 72 h. These figures show increases in the initial current that resulted from the dissolution of the oxide from the surface; this oxide must have formed either in the air before the measurement or during immersion in the SBF solution. After a few minutes, the current gradually decreases as the alloys develop a thick corrosion product and/or oxide layer with time [37,39,41].

During measurement, the largest absolute values for current were recorded for the alloy that has only four Sn added (curve 1). The alloys also displayed some fluctuations, indicating a higher rate of corrosion not only uniform but also pitting, which rarely occurs because the highest absolute current was in the range of some microamperes, particularly after a long time of the application of the applied potential, >30 min [41]. Increasing the Sn content to 6% significantly reduced the value of the current over time, and more reduction was recorded when the content of Sn was increased to 8%. This is because an increase in Sn% reduces general corrosion and prevents pitting attack [29,37]. The CCt behavior seen in Figure 4, which is for the different alloys after a 72-h exposure, recorded a similar behavior to that seen in Figure 3 (CCt curves after only one hour), and the only difference was a high reduction in the current values. Thus, the addition of Sn increases the corrosion resistance and eliminates pitting corrosion, and this effect becomes clearer as time is increased to 72 h.

### 3.3. SEM and EDX Analysis

**Figure 5:**
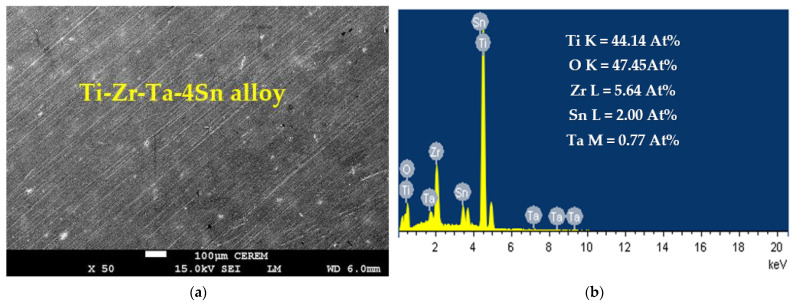
(**a**) SEM and (**b**) EDX analyses for Ti-Zr-Ta-4Sn alloy that was submerged in the SBF solution for 72 h.

To show the surface morphology and composition of the layers formed due to the corrosion of the alloys at high anodic potential after a 72-h immersion in the SBF solution, we utilized SEM and EDX analyses. Ti-Zr-Ta-4Sn alloy’s SEM micrograph and EDX are demonstrated in Figure 5, where, although there may be a few tiny pits, the surface does not exhibit significant uniform corrosion. The CCt experiment, which corresponds to the same alloy under the same conditions and has demonstrated some variations in the currents over time, is shown in Figure 4 (curve 1). The following components were found using EDX analysis on the surface of the alloy under test: 44.14% Ti, 47.45% O, 5.64% Zr, 2.00% Sn, and 0.77% Ta. The high weight percentage of Ti and O suggests that the surface forms a thick coating of TiO_2_ and that the coating layer is the reason for the lower recorded current values under these circumstances (Figure 4, curve 1). This allows the protection of the alloy in the SBF solution. Additionally, the fact that Zr, Sn, and Ta concentrations are lower than anticipated compared to their original concentrations (15%, 4%, and 2%, respectively) further supports the existence of a TiO_2_ oxide layer [37,41].

**Figure 6:**
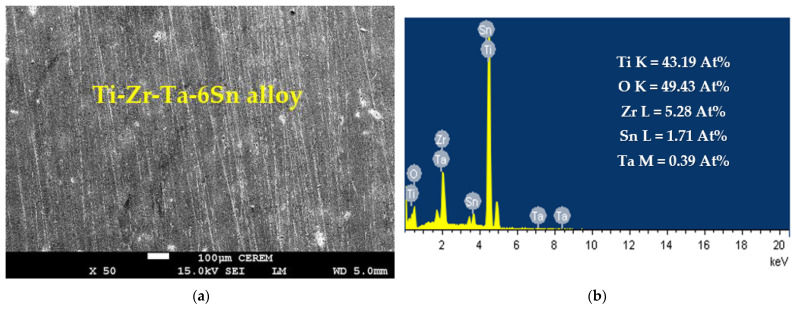
(**a**) SEM and (**b**) EDX analyses for Ti-Zr-Ta-6Sn alloy that was submerged in SBF solution for 72 h.

**Figure 7:**
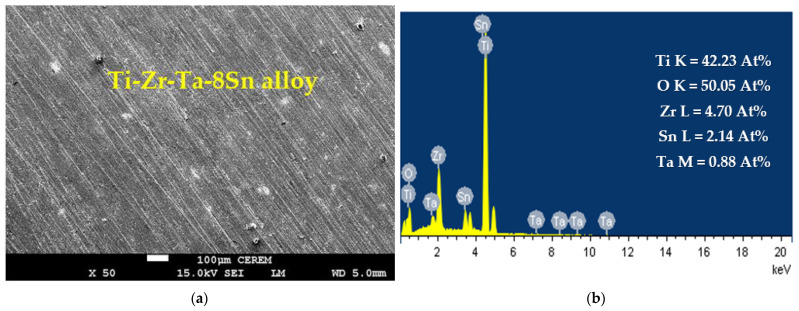
(**a**) SEM and (**b**) EDX analyses for Ti-Zr-Ta-8Sn alloy that was submerged in SBF solution for 72 h.

A similar SEM micrograph and EDX profiling for the alloy with 6% Sn were also taken, as shown in Figure 6. The image of this alloy shows that the surface does not show either uniform or pitting corrosion. This again supports the CCt experiment shown in Figure 7 (curve 2). The EDX profile also indicates that the surface of the alloy contained 43.19% Ti, 49.43% O, 5.28% Zr, 1.71% Sn, and 0.39% Ta. It is understood that Ti and O have the highest content, which is due to a considerably thick layer of TiO_2_ that was formed [39]. The presence of TiO_2_ decreases the obtained currents and leads to more passivation of the surface of the tested Ti-base alloy. It is also worth mentioning that the percentages of Zr, Sn, and Ta are lower than their original percentages within the alloy, indicating that the formed TiO_2_ layer does not allow the appearance of the other component of the alloy.

The effect of increasing the Sn content to 8% on the surface morphology and its profile were achieved, as shown in Figure 7. The image confirms that the surface is clean and does not have any pits. It is seen also in the SEM image that uniform corrosion does not occur due to the protection of the surface by increasing the content of Sn to 8%. The elements obtained on this surface are 42.23% Ti, 50.05% O, 4.70% Zr, 2.14% Sn, and 0.88% Ta. Again, the presence of these elements with their detected percentages confirms that the surface forms a thick TiO_2_ film, which is thicker than the ones obtained on the surface of the alloys that have only 4% Sn (Figure 5) and 6% Sn (Figure 6), thus leading to a decrease in the obtained current, as can be seen from Figure 4 (curve 3). The CCt along with SEM and EDX analyses prove that magnifying the Sn content from 4% to 6% and further to 8% considerably decreases the recorded currents with time at 400 mV, and this effect significantly reduces the corrosion of Ti-Zr-Ta-Sn alloys in the SBF solution.

### 3.4. EIS Results

**Figure 8:**
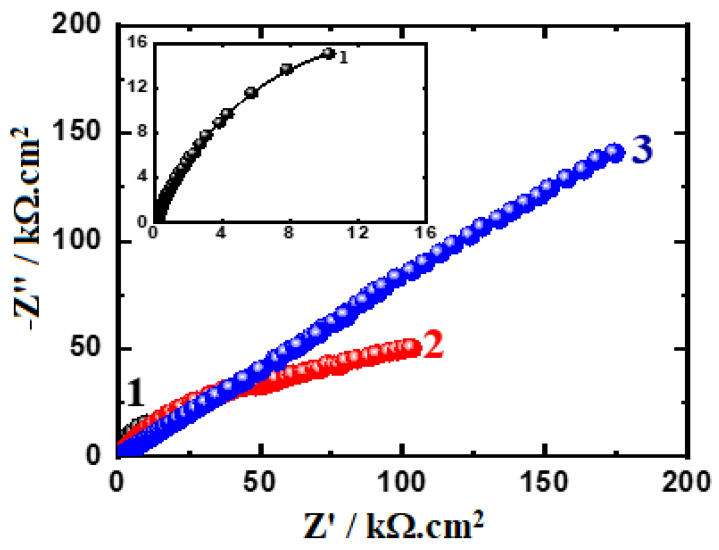
Nyquist graphs for (1) Ti-Zr-Ta-4Sn, (2) Ti-Zr-Ta-6Sn, and (3) Ti-Zr-Ta-8Sn in SBF after a one-hour immersion.

**Figure 9:**
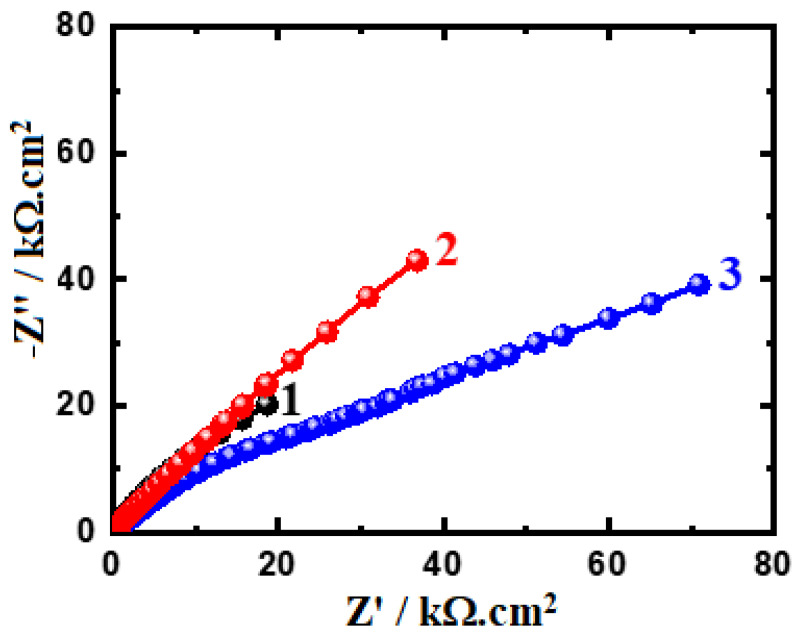
Nyquist graphs for (1) Ti-Zr-Ta-4Sn, (2) Ti-Zr-Ta-6Sn, and (3) Ti-Zr-Ta-8Sn in SBF after a 72-h immersion.

**Figure 10:**
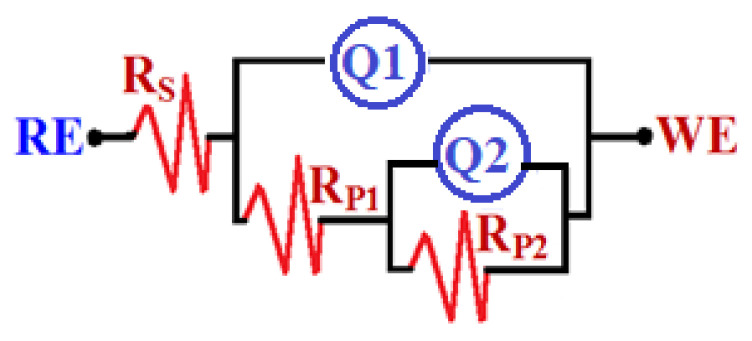
The equivalent circuit model that best fits the impedance data.

The EIS method has the ability to explain the reactions for the transfer of electrons at the interface of the Ti-Zr-Ta-Sn developed alloys and the SBF solution. The impedance technique has been utilized to investigate and report the phenomena of corrosion and its passivation under different experimental conditions [42,43,44,45]. Figure 8 displays the Nyquist spectra for the alloys (1) Ti-Zr-Ta-4Sn, (2) Ti-Zr-Ta-6Sn, and (3) Ti-Zr-Ta-8Sn in SBF solution for one hour. The Nyquist spectra after a 72-hour exposure are also plotted, as seen in Figure 9. The data presented in Figure 8 and Figure 9 were fitted to the circuit model shown in Figure 10. It is worth confirming that the equivalent circuit model shown in Figure 10 has been cited in many research studies [33,41,44]. The elements of the circuit are R_S_ as the solution resistance, Q_1_ and Q_2_ as the first and second constant phase elements, and R_P1_ and R_P2_ as the first and second polarization resistances [39,41].

The Nyquist plots display one semicircle in which its diameter has the smallest area recorded for the alloy with 4% Sn (Ti-Zr-Ta-4Sn alloy). Increasing the Sn content to 6% led to increasing the diameter of the semicircle, and this effect is increased when the Sn content in the alloy is 8%. After 72 h, the size of the obtained semicircles decreased, as seen for the alloys with 6% Sn and 8% Sn (Figure 9). Extending the exposure time to t, 72 h indicates that the resistance to corrosion for the tested alloys decreases as a result of the increased attack by the corrosive ions in the solution. Thus, the Nyquist plots indicate that the increase in Sn from 4% to 8% significantly increases the resistance of the Ti-base alloy towards corrosion. On the other hand, increasing the time of exposure to 72 h reduces the overall resistance to corrosion when compared to the ones recorded for the alloys after a one-hour immersion.

The data recorded in Table 2 confirmed that R_S_ increases as Sn % goes up and that this effect also increases R_P1_ and R_P2_. Here, R_P1_ is considered the resistance that is presented on the alloys’ surface and the formed film of corrosion products on their surface. In addition, the second polarization resistance, R_P2_, is the resistance in the outer interface of the surface, forming the corrosion product and electrolyte. The increase in the values of these resistances with an increasing percentage of Sn reveals that increasing Sn% improves the corrosion resistance as a result of increasing the passivation of the alloys against corrosion.

Furthermore, the presence of the 1st phase constant phase elements, Q_1_, represents a weak double-layer capacitor. This is because the values of the component “n” that accompany Q_1_ range from 0.83 and 0.68. This is reflected in the Y_Q1_ values, which decrease with increasing Sn% either with short or long immersion times, indicating that the passivation of the surface of the alloys was enhanced with increasing Sn content. The presence of the second constant phase element, Q_2_, which can also be regarded as another double-layer capacitor due to the values of “n” ranging from 0.92 to 0.84, further confirmed this impact [39,41]. The existence of Q in such a capacitive response system has reportedly been used to gauge departures from ideality, as has been reported by Diamanti et al. [44]. Thus, the EIS findings concur with the results obtained from the CPP experiments, and both verified that the corrosion resistance increases noticeably as the Sn content rises from 4% to 8%, although the same sequence is obtained with lower values when the immersion time is extended to 72 h.

## 4. Conclusions

Ti-Zr-Ta alloys with different Sn contents were manufactured by mixing their pure powders in the required percentages. The corrosion resistance of the synthesized Ti-Zr-Ta-xSn alloys was studied using the CPP, CCt, and EIS measuring techniques after two exposure periods, one hour and 72 h. The results of all test techniques indicate that adding Sn to the Ti-base alloy increases the corrosion resistance, and the effect increases with the increase in Sn content to reach its maximum for the alloy that has 8% Sn. Here, 8% Sn significantly reduces the corrosion current (j_Corr_) measured via the CPP technique and thus the corrosion rate that was calculated from the values of j_Corr_. The experiments of CCt prove that the absolute currents decrease with increasing Sn content, which means that the severity of corrosion decreases. The EIS data also confirmed that Ti-Zr-Ta-8Sn has the widest diameter, as shown by the Nyquist plots. Both CPP and EIS results also revealed that prolonging the exposure time slightly increases j_Corr_ and consequently decreases R_P_. Furthermore, surface (SEM and EDX) investigations were performed after measuring CCt for all alloys. These SEM and EDX analyses proved that the Ti-Zr-Ta-4Sn highly resists corrosion, which is further enhanced when the content of Sn is magnified to 8%. Thus, all data confirmed that increasing the Sn content greatly allows the alloys to resist corrosion in the SBF solution and that prolonging the exposure time enhances this effect.

## Figures and Tables

**Table 1:** Polarization data for the various alloys in SBF solution.

Alloy/Exposure Time	βc (mV/dec)	βa (mV/dec)	E_Corr_ (mV)	j_Corr_ (μA/cm^2^)	R_P_ (KΩ.cm^2^)	R_Corr_ (mm/yr)
Ti-Zr-Ta-4Sn/one hour	130	140	−540	0.28	112.44	0.00244
Ti-Zr-Ta-6Sn/one hour	120	130	−380	0.18	150.72	0.00157
Ti-Zr-Ta-8Sn/one hour	120	130	−315	0.05	542.61	0.00044
Ti-Zr-Ta-4Sn/72 h	140	160	−680	0.35	92.75	0.00305
Ti-Zr-Ta-6Sn/72 h	130	150	−380	0.25	121.12	0.00218
Ti-Zr-Ta-8Sn/72 h	120	130	−210	0.14	193.79	0.00122

**Table 2:** Parameters were determined by fitting the EIS data for the various alloys.

Sample	Impedance Data						
	R_S_/ Ω cm^2^	Q_1_		R_P1_/ Ω cm^2^	Q_2_		R_P2_/ Ω cm^2^
		Y_Q1_/F cm^−2^	n		Y_Q2_/F cm^−2^	n	
Ti-Zr-Ta-4Sn/one hour	78.6	0.0042	0.83	9306	0.0047	0.89	9590
Ti-Zr-Ta-6Sn/one hour	110.1	0.0025	0.81	34,470	0.0038	0.87	36,542
Ti-Zr-Ta-8Sn/one hour	155.1	0.0019	0.75	128,978	0.0024	0.84	62,587
Ti-Zr-Ta-4Sn/72 h	135.9	0.0088	0.86	9829	0.0064	0.87	8982
Ti-Zr-Ta-6Sn/72 h	151.8	0.0075	0.79	19,523	0.0047	0.85	24,581
Ti-Zr-Ta-8Sn/72 h	164.1	0.0049	0.68	31,741	0.0036	0.92	48,395

## Data Availability

Not applicable.
